# Response: Commentary: The energy model of insulin resistance: a unifying theory linking seed oils to metabolic disease and cancer

**DOI:** 10.3389/fnut.2025.1690496

**Published:** 2025-10-02

**Authors:** Catherine Shanahan

**Affiliations:** Rebel Well, LLC, Orlando, FL, United States

**Keywords:** insulin resistance, bias, oxidative stress, metabolic disease, seed oil, mitochondria, paradigm (gestalt) shift, cancer

Lopez-Moreno is correct to question the hypothesis presented. After all, it has not yet been directly tested. But it can be tested—and it should be. That is the purpose of advancing a new mechanistic model: to generate hypotheses that can guide future research, challenge entrenched assumptions, and refine our understanding of disease processes.

Furthermore, I appreciate his hesitancy to accept a hypothesis that begins by questioning the accuracy of decades of nutrition ideology. However, rather than adopting a posture of scientific inquiry, the reviewer instead appears intent on shutting down further discussion.

Consider, for example, his comments on Figure 1 in the original article (1). Then cite the article as reference 1. The figure presents a basic yet essential observation: over the past century, RBD seed oil intake has increased dramatically and in parallel with the rise in type 2 diabetes, while other macronutrients, including carbohydrates, meat, and saturated fat, show no such parallels. This is not presented as proof of causality, but as a necessary starting point—a shared empirical reality that demands attention.

Yet instead of engaging with the data, López-Moreno critiques (Figure 1) for misrepresenting correlation as causation. This framing is egregiously inaccurate. It implies that the figure is intended as a stand-alone argument linking seed oil consumption to metabolic disease. In truth, its purpose is far more modest and foundational: to establish the historical dietary context in which rising rates of metabolic disease have occurred.

To dismiss this correlation as a fallacy, without engaging with the broader context of the article, is to mischaracterize the figure entirely. It is neither the conclusion of the argument nor the evidence upon which the model rests. It simply invites the reader to ask: why is the correlation between seed oil consumption and diabetes so rarely acknowledged in mainstream discourse, especially when other presumed culprits fail even to correlate?

López-Moreno also overlooks a critical and well-documented dataset discussed in the article: that dietary seed oils have profoundly altered human adipose tissue composition over recent decades. This shift is supported by both observational and interventional data, and has implications for mitochondrial function, oxidative stress, and insulin signaling.

To disregard this finding, or attempt to dismiss it as irrelevant or coincidental, is not a responsible act of scientific skepticism. It reflects a refusal to engage with emerging lines of evidence that challenge long-standing assumptions. In an era of rising chronic disease and widespread dietary confusion, such reflexive dismissal is not just unscientific—it is part of the problem.

As further evidence that the reviewer intends to misrepresent the scientific argument presented, he incorrectly frames my hypothesis as a rebranding of the carbohydrate-insulin model (CIM). It should be abundantly clear that the energy model diverges fundamentally from CIM, which focuses on dietary carbohydrates and insulin while the energy model centers on how redox imbalance brought about by chronic seed oil consumption might impair the cell's oxidative metabolic capacity and create an increased reliance on glycolysis. This has nothing to do with dietary carbohydrates or insulin.

The reviewer also falsely claims I present no human evidence for my hypothesis when in fact the article cites two of the longest, most rigorous RCTs ever conducted—The Minnesota Coronary Experiment and The Sydney Diet Heart Study—both of which showed increased mortality in the seed oil substitution arms. Additionally, I include citations to human mechanistic studies linking RBD seed oils to lipid peroxidation, altered mitochondrial function, and insulin resistance.

Throughout the commentary, the reviewer falls back on a familiar set of rhetorical strategies. He recasts peer-reviewed toxicity data from Martin Grootveld as “alarmist rhetoric.” He dismisses non-human toxicological evidence as irrelevant—even though toxicology research is necessarily conducted in animals due to ethical constraints on human testing. He claims I dismiss “decades of research and expert consensus” when in fact I merely suggest that we revisit that consensus in light of substantial evidence that has been overlooked or ignored. He invokes an invisible army of “esteemed organizations” like the AHA and ADA as the ultimate arbiters of truth, but ignores the evidence I presented that the AHA promoted seed oil consumption before any human trials were conducted. When later RCTs such as the Minnesota Coronary Experiment and Sydney Diet Heart Study revealed increased mortality in the intervention groups, these findings were ignored or buried. Highlighting this pattern is not “alarmist.” It is a call for scientific accountability and methodological rigor.

Taken together, these errors raise a broader concern: the potential for ideological framing to shape scientific interpretation. The reviewer is affiliated with a group that promotes plant-based diets for sustainability reasons. While he does not cite sustainability directly, institutional ideology can influence whether new ideas are accepted or dismissed. It is essential that metabolic health research remain grounded in physiological evidence—not broader political or environmental narratives—so that public health guidance reflects scientific rigor rather than advocacy.

In closing, the energy model of insulin resistance is not a product of a “trend” of making “unsubstantiated claims.” Nor is it “alarmist” speculation. It is a testable mechanistic framework that integrates biochemical, clinical, and historical data to explain persistent patterns of metabolic dysfunction that current models do not adequately address. The reveiwer's mischaracterizations, reliance on consensus appeals, and omission of relevant contradictory evidence underscore the need for rigorous, evidence-based re-examination of all potential contributors to the chronic disease epidemic—including the long-overlooked role of RBD seed oils.

This reviewer's commentary is not just about his disagreements with my particular ideas. When dissenting hypotheses are reflexively labeled “alarmist” and excluded from serious discourse, the scientific community risks silencing insights that could reshape our understanding of chronic disease. What are we overlooking by stifling challenges to the status quo? How many promising research directions have been prematurely closed off—not because they were disproven, but because they provoked cognitive dissonance? In an era of escalating chronic disease, we must be willing to dive into this dissonance rather than turn away from the discomfort it tends to provoke. We do this by revisiting foundational assumptions with openness, rigor, and a clear-eyed commitment to truth as our north star.

## References

[1] Shanahan C. The energy model of insulin resistance: A unifying theory linking seed oils to metabolic disease and cancer. Front. Nutr. (2025) 12:1532961. 10.3389/fnut.2025.1532961 40421040 PMC12105547

